# Hospital Cookery

**Published:** 1924-11

**Authors:** 


Sir,—The plaint of " A La Lanterne," in your last issue, as to the want of kindliness—I had almost written of humanity—displayed in catering for the sick in our hospitals is a timely one. It is, of course, difficult to know how best to move in the matter, because the hot mutton, potatoes, and cabbage, followed by rice pudding, which, year in year out, are supplied to hospital patients has apparently become as much a matter of tradition in a hospital as that a nurse should wear a stiff collar and have her uniform dress made with a waist.

But there is at least one point which can be altered by those in charge of the wards, without any printed rule or any expense—namely, to fit the portion served to the patient for whom it is intended. If the mechanic has a broken leg only and is otherwise perfectly fit, by all means pile his plate to overflowing; he will enjoy it. But why do just the same to the poor little gentlewoman who has abdominal trouble and is nervy and has no appetite whatever ? The result is disastrous in two ways. The patient sickens at the sight of the plethora, and eats nothing ; the hospital finances suffer, for all the discarded food is thrown away.

Common Sense.

